# Quality of Security Guarantees for and with Physical Unclonable Functions and Biometric Secrecy Systems †

**DOI:** 10.3390/e25081243

**Published:** 2023-08-21

**Authors:** Onur Günlü, Rafael F. Schaefer, H. Vincent Poor

**Affiliations:** 1 Information Coding Division, Linköping University, 581 83 Linköping, Sweden; 2 Chair of Information Theory and Machine Learning, BMBF Research Hub 6G-life, Cluster of Excellence “Centre for Tactile Internet with Human-in-the-Loop (CeTI)”, and 5G Lab Germany, Technische Universität Dresden, 01062 Dresden, Germany; rafael.schaefer@tu-dresden.de; 3 Department of Electrical and Computer Engineering, Princeton University, Princeton, NJ 08544, USA; poor@princeton.edu

**Keywords:** quality of security (QoSec), physical unclonable function (PUF), reliability on the quantization boundary, transforms without multiplications, IoT security

## Abstract

Unique digital circuit outputs, considered as physical unclonable function (PUF) circuit outputs, can facilitate a secure and reliable secret key agreement. To tackle noise and high correlations between the PUF circuit outputs, transform coding methods combined with scalar quantizers are typically applied to extract the uncorrelated bit sequences reliably. In this paper, we create realistic models for these transformed outputs by fitting truncated distributions to them. We also show that the state-of-the-art models are inadequate to guarantee a target reliability level for all PUF outputs, which also means that secrecy cannot be guaranteed. Therefore, we introduce a quality of security parameter to control the percentage of the PUF circuit outputs for which a target security level can be guaranteed. By applying the finite-length information theory results to a public ring oscillator output dataset, we illustrate that security guarantees can be provided for each bit extracted from any PUF device by eliminating only a small subset of PUF circuit outputs. Furthermore, we conversely show that it is not possible to provide reliability or security guarantees without eliminating any PUF circuit output. Our holistic methods and analyses can be applied to any PUF type, as well as any biometric secrecy system, with continuous-valued outputs to extract secret keys with low hardware complexity.

## 1. Introduction

Device identification and authentication help in protecting sensitive data. Similar to identifying a person by using their biometric identifiers, one can use physical identifiers, e.g., physical unclonable functions (PUFs) [1,2,3], to reliably and uniquely identify a digital device that embodies the physical identifier. For instance, one can secure internet-of-things (IoT) devices that carry private data by using PUFs that are embodied by these devices, for which secret keys (SKs) can be extracted from PUF outputs, such that one can reliably reconstruct the SK on demand [4]. The extracted SK can be considered as a root of trust that is hardware-intrinsic and suitable for applications in cryptography, intellectual property protection, sensor identification/authentication, etc. [5,6]. Therefore, digital circuit outputs that are reliable and high-entropy, such as ring oscillator (RO) frequencies, can be used as PUFs that are cheaper and safer alternatives to using nonvolatile memory to store SKs [7,8,9]. Note that PUFs are safer because SK reconstruction takes place on demand, and invasive attacks to the hardware change the digital circuit outputs permanently, which help to eliminate the necessity of costly uninterrupted hardware protection [10].

Mainly because there are random temporal variations in the hardware, digital circuit output measurements are noisy. Furthermore, mainly because surrounding logic circuitry causes systematic variations in the digital circuit outputs, different digital circuit outputs embodied in the same device are correlated [7,11]. The noise causes errors in the reconstructed SK, which can be corrected by using error correcting codes (ECCs) [7]. Moreover, PUF output symbol correlations may increase the amount of SK information leaked to an eavesdropper who has side information about the correlations. This follows as the eavesdropper can then apply machine learning algorithms for modeling the PUF outputs [12,13]. When the noise components in the PUF measurements are additive, simple SK agreement schemes that apply ECCs, called *helper data schemes*, can be used. Two classic examples of such schemes are code-offset fuzzy extractors [14] and the fuzzy commitment scheme (FCS) [15], which are extended in [16,17] under a constraint on the amount of helper data. These schemes, however, require that PUF outputs are uniformly distributed and independent and identically distributed to achieve the SK capacity [18,19,20,21]. Therefore, transform coding methods are proposed in [12,22,23] to decorrelate the PUF circuit outputs such that the transformed outputs are quantized via uniform scalar quantizers, which allows one to extract almost uniformly distributed and independent and identically distributed outputs; see [24,25,26] for their applications to biometric identifiers and [27,28] for alternative methods.

### 1.1. Summary of Contributions

Consider correlated and noisy PUF circuit output symbols that are realizations of a random variable with a continuous alphabet, such as for RO PUFs. We extract SKs from such PUFs by applying a new transform coding method that improves on the state-of-the-art methods. Toward this aim, we (i) model noiseless transform coefficients that are obtained from noiseless PUF circuit outputs as random variables with a truncated probability distribution to take account of the fact that most digital circuit output measurements are realizations of a finite set; (ii) introduce a quality of security (QoSec) parameter that refers to the PUF output percentage for which one can guarantee target reliability and security levels. Moreover, we characterize how the QoSec parameter affects the tradeoff between the average or maximum error probability and the number of bits extracted from transform coefficients; and (iii) prove that there are significantly better schemes than the two mentioned helper data schemes, which follows by showing that the model for the measurement channel is generally not memoryless.

In addition to the contributions mentioned above that are provided in the conference version of this work in [29], we have the following further significant contributions.

- We propose a joint thresholding approach to provide QoSec guarantees under constraints on the SK size and block error probability to achieve (secret-key, privacy-leakage) rate tuples that are close to the finite-length information-theoretic bounds on the rate region boundary;
- We apply our proposed approach to an RO output dataset to illustrate the effects of different QoSec values on the manufacturing yield and the SK size for a small number of ROs, while using state-of-the-art transforms that are orthogonal and that can be computed without multiplications. This result shows that providing QoSec guarantees do not cause a significant performance degradation.

### 1.2. Notation

We denote random variables with upper case letters *X* and their realizations with lower case letters *x*. A sequence of random variables is denoted as $X^{n}=X_{1},\ldots ,X_{i},\ldots ,X_{n}$, where a subscript *i* denotes the position of a variable in the string. A random variable X has a probability mass function $P_{X}$ or probability density function $f_{X}$. The sets are represented by calligraphic letters X with size |X|. Enc(·) is an encoder mapping, and Dec(·) is a decoder mapping. The function $H_{b}\left(q\right)=-q\log q-\left(1-q\right)\log\left(1-q\right)$ denotes the binary entropy function, and all logarithms in this work are natural logarithms. O(·) denotes the big *O* notation. Q(·) is the Q-function, and $Q^{-1}\left(\cdot \right)$ its inverse. *I* denotes the identity matrix, and *T* is the matrix transpose. The operator ⊕ represents the element-wise modulo-2 summation. [a:b] denotes the set of integers a,(a+1),…,b. A binary symmetric channel (BSC) with crossover probability *p* is denoted by BSC(*p*) for p∈(0,1).

### 1.3. Organization

This paper is organized as follows. In Section 2, we describe the output model for ROs and the transforms that are applied to design RO PUFs. In Section 3, we discuss the SK agreement with PUFs and provide the asymptotic and non-asymptotic limits for the tradeoffs between reliability, secrecy, and privacy to argue for the FCS that achieves asymptotic optimality at a particular point on the rate region. In Section 4, we propose a new transform coding method. In Section 5, we impose a QoSec constraint and define the performance metrics to be used for comparisons. In Section 6, we propose a novel joint thresholding approach and illustrate the effects of providing QoSec guarantees by applying the proposed transform coding method and the joint thresholding approach to a public RO output dataset. In Section 7, we conclude the paper.

## 2. Model for RO Outputs

A classic PUF type is the RO PUF, which has positive- and continuous-valued outputs. We describe the digital circuit model for ROs and focus on them in this work. However, the same analyses can be applied to all PUF types with continuous-valued outputs.

**Figure 1:**
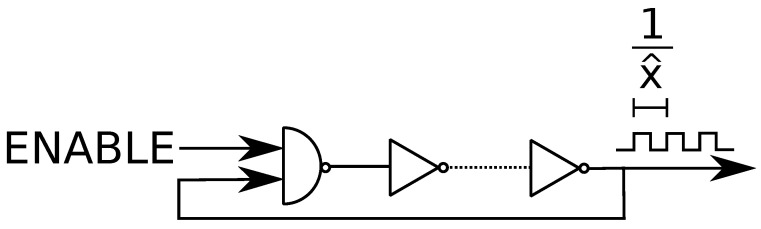
The RO logic circuit.

The RO logic circuit consists of an odd number of inverters that are serially connected, where the final inverter output is fed back to the first inverter; see Figure 1. In general, a NAND gate is used from the first gate, since it allows one to disable the RO when it is unused, and since it has the same logic output as an inverter otherwise. Uncontrollable manufacturing variations change the total propagation delay of a signal that flows through an RO, which can be equivalently described as the oscillation frequency $\hat{x}$. If we have (2m+1) inverters, for any m≥1, in the RO logic circuit, then we obtain [30]

(1) $$\hat{x}=\frac{1}{2\left(2m+1\right)\tau_{d}},$$

where $\tau_{d}$ represents the propagation delay governed by the circuitry nonlinearities and parasitics, which are mainly uncontrollable. Therefore, RO oscillation frequencies are suitable to be used as a local source of randomness.

Because of the random noise sources, such as thermal noise and flicker noise, RO measurement outputs are noisy. Moreover, because of the deterministic effects, including the surrounding logic circuitry and cross talk between adjacent signal traces, RO outputs in the same device are correlated [9,10,30]. A simple binary SK extraction method is to apply hard decisions to RO output pairs to put out a bit by comparing the oscillation frequencies [7]. This simple method is not secure since the dependency between different RO outputs in the same device, as discussed above, results in extra secrecy leakage [31]. Thus, in [22], a discrete cosine transform (DCT)-based transform coding scheme was applied, which reduced the dependencies before applying scalar quantizers. Furthermore, in [12], the discrete Walsh Hadamard transform (DWHT) was shown to achieve a similar decorrelation performance as the DCT, while requiring a smaller hardware area. As an extension of the DWHT, a new set of orthogonal transforms was shown in [23] to further decrease the bit error probability without increasing the hardware area as compared to the DWHT. In this work, we use the new set of orthogonal transforms proposed in [23] and then publicly select the best transform in the set that has the highest decorrelation efficiency and reliability performance with QoSec guarantees.

## 3. Fuzzy Commitment Scheme for SK Agreement

Consider that we want to reconstruct a predetermined SK on demand by using PUF outputs without leaking information about the SK. One can achieve this by using ECCs by correcting the errors in the noisy PUF digital circuit outputs [19,21]. The FCS is a powerful method that uses a masking (i.e., one-time padding) step and an error correction step to reliably reconstruct an SK by using the noisy measurements of digital circuit outputs that are used as PUFs. Suppose, without loss of generality, that the first PUF output measurement is noiseless although other measurements are noisy, which can be directly extended to a hidden PUF source model with noisy first measurements, as discussed in [17] [Appendix B]. The *n*-letter sequence $X^{n}\in X^{n}$ denotes the first PUF output measurement. We embed a predetermined SK S∈S to bind $X^{n}$ to *S* in such a way that the output of the binding operation and the second PUF output measurement $Y^{n}\in Y^{n}$ suffice to reliably reconstruct the SK *S*. The output of the binding operation is denoted as $W^{n}\in W^{n}$, which is called helper data, and is obtained via a masking step that adds two *n*-letter sequences. Suppose a linear ECC C has blocklength *n*, code dimension log|S|, encoder Enc(·), and decoder Dec(·). The FCS masking step computes the sum of a codeword $C^{n}$ and $X^{n}$ in modulo-|X|, in which the codeword is computed by encoding the SK *S*, i.e., $C^{n}=\mathrm{Enc}\left(S\right)$. Assume for simplicity that X=Y=W={0,1}, so one can use a binary linear ECC. Then, we obtain $W^{n}=X^{n}⊕C^{n}$, which is the helper data available in the hardware publicly. We can represent the second PUF measurement output $Y^{n}$ as $Y^{n}=X^{n}⊕E^{n}$, where $E^{n}\in {\left\{0,1\right\}}^{n}$ represents a binary error sequence. Thus, we have $W^{n}⊕Y^{n}=C^{n}⊕E^{n}$, which allows the decoder Dec(·) to reliably map $C^{n}⊕E^{n}$ into an index $\hat{S}\in S$ that is equal to the predetermined SK *S* with high probability.

**Figure 2:**
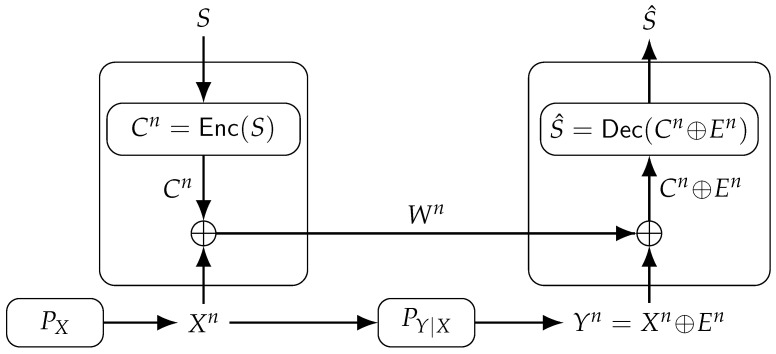
Fuzzy commitment scheme (FCS).

We depict the FCS in Figure 2, where $X^{n}$ is assumed to follow an independent and identically distributed Bernoulli distribution $P_{X}$, and the channel $P_{Y^{n}|X^{n}}$ is assumed to be memoryless, i.e., $P_{Y^{n}|X^{n}}=P_{Y|X}^{n}$. To achieve an optimal rate tuple on the rate region boundary by using the FCS, these assumptions are necessary but not sufficient [15]. We next define all (secret-key, privacy-leakage) rate pairs that can be achieved by using the FCS under secrecy, reliability, and privacy constraints.

**Definition** **1.** *A (secret-key, privacy-leakage) rate pair $\left(R_{s},R_{\ell }\right)$ is called* achievable *by using the FCS if, for any ϵ>0, there exist n≥1, an encoder Enc(·), and a decoder Dec(·) that satisfy*

(2) $$\begin{array}{cccc} & P_{e}=\mathrm{Pr}\left[\hat{S}\neq S\right]\leq \epsilon & & \;(\mathrm{reliability}) \end{array}$$

(3) $$\begin{array}{cccc} & \frac{1}{n}H\left(S\right)\geq R_{s}-\epsilon =\frac{\log|S|}{n}-\epsilon & & \;(\mathrm{SK}\;\mathrm{uniformity}) \end{array}$$

(4) $$\begin{array}{cccc} & I\left(S;W^{n}\right)=0 & & \;(\mathrm{zero}\;\mathrm{secrecy}\;\mathrm{leakage}) \end{array}$$

(5) $$\begin{array}{cccc} & \frac{1}{n}I\left(W^{n};X^{n}\right)\leq R_{\ell }+\epsilon \;\;\;\; & & \;(\mathrm{privacy}\text{-}\mathrm{leakage}\;\mathrm{rate}). \end{array}$$

The reliability constraint in (2) requires that the SK *S* should be reconstructed reliably with a negligible probability of error. The SK uniformity constraint in (3) requires the SK *S* with rate $R_{s}$ to be almost uniformly distributed. The zero secrecy leakage constraint in (4) requires that the helper data $W^{n}$, which is the only public sequence, should not leak any information to an eavesdropper about the SK *S*, i.e., perfect secrecy is achieved. Finally, the privacy-leakage rate constraint in (5) requires the normalized information leakage about $X^{n}$ to an eavesdropper to be not nonnegligibly larger than $R_{\ell }$. Such a privacy leakage constraint is imposed because if there are multiple PUF enrollments of the same PUF, we observe extra secrecy leakage when the privacy leakage is high. Note that in general, the unnormalized privacy leakage $I(W^{n};X^{n})$ is unbounded unless there is a private key available [18], which is not necessarily a realistic assumption, since if one can keep a private key hidden from an eavesdropper, then there is no need to bind SKs to PUF outputs [32].

The measurement channel $P_{Y|X}$ for PUFs is generally modeled as a BSC(p), which applies, e.g., to static random access memory PUFs [6]. We illustrate in the next section that a BSC fits well as the channel model also for PUF circuits with continuous-valued outputs, e.g., RO PUFs, when transformation is followed by uniform scalar quantizers and when the transform coefficients and all noise components follow symmetric probability distributions. The decorrelation performance of a transform is one of the criteria for choosing the orthogonal transform to apply to PUF circuit outputs, since the FCS requires almost independent and identically distributed PUF output symbols that are obtained after quantization. Moreover, the FCS analysis assumes that the measurement channel $P_{Y^{n}|X^{n}}$ is memoryless. We show in the next section that after applying the new transform coding method to RO PUFs, we have PUF measurements $X^{n}$ that are almost independent and identically distributed and that follow a binary uniform distribution, while we have the measurement channel $P_{Y^{n}|X^{n}}≃\prod_{i=1}^{n}P_{Y_{i}|X_{i}}$, where $P_{Y_{i}|X_{i}}$ is a BSC(p) for all i∈[1:n]. We next provide the rate region for this model.

**Theorem** **1** ([20])**.** *If $X^{n}$ is independent and identically distributed, $P_{X}$ is a binary uniform distribution, and $P_{Y|X}^{n}$ is a ${\mathrm{BSC}}^{n}\left(p\right)$, the rate region of all rate pairs $\left(R_{s},R_{\ell }\right)$ achievable by using the FCS is*

(6) $$\begin{array}{cc} \{\left(R_{s},R_{\ell }\right)\;:\;\; & 0\leq R_{s}\leq 1-H_{b}\left(p\right),\\ \; & R_{\ell }\geq 1\;-\;R_{s}\}. \end{array}$$

Note that the only tuple that is achievable by using the FCS and that is asymptotically optimal is $\left(R_{s}^{*},R_{\ell }^{*}\right)=\left(1\;-\;H_{b}\left(p\right),H_{b}\left(p\right)\right)$[18,20], in which $R_{s}^{*}$ is equal to the channel capacity of $P_{Y|X}$. Thus, maximizing the code rate of the ECC C suffices to achieve asymptotically optimal rate tuples for the FCS. Moreover, the FCS treats reliability and security separately [20]; so, one can directly apply the finite-length results for reliable communications to the rate region of the FCS. Therefore, we have the following accurate approximation for the finite-length rate region of the FCS that approximates both achievability and converse bounds.

**Theorem** **2** (Normal Approximation [33])**.** *Given an n-letter independent and identically distributed $X^{n}$ that is distributed according to a binary uniform distribution $P_{X}$, a memoryless measurement channel $P_{Y|X}$ that is a BSC(p), and a block error probability $P_{e}$, there exist rate pairs $\left(R_{s},R_{\ell }\right)$ that are achievable by using the FCS such that*

(7) $$\begin{array}{cc} \; & R_{s}\left(n,p,P_{e}\right)=C\left(p\right)-\sqrt{\frac{V(p)}{n}}Q^{-1}\left(P_{e}\right)+0.5\frac{\log(n)}{n}+O\left(\frac{1}{n}\right), \end{array}$$

*where we have*

(8) $$\begin{array}{cc} \; & C\left(p\right)=\log\left(2\right)-H_{b}\left(p\right), \end{array}$$

(9) $$\begin{array}{cc} \; & V\left(p\right)=p\left(1-p\right)\log^{2}\left(\frac{1-p}{p}\right) \end{array}$$

*and*

(10) $$\begin{array}{c} R_{\ell }\geq 1-R_{s}. \end{array}$$

We next illustrate how to provide QoSec guarantees by proposing a new transform coding method. Then, we illustrate, by using Theorem 2, accurate approximations for the finite-length rate regions for SK agreement with FCS for an available PUF output dataset under a realistic block error probability $P_{e}$ constraint to analyze the effects of the QoSec guarantees on practical setups.

## 4. New Transform Coding Steps

We propose a transform coding method that is suitable for any continuous-valued biometric and PUF outputs, and we analyze the performance of RO PUFs when the proposed transform coding method is applied. Consider that we implement r≥1 ROs as a two-dimensional (2D) array that has size $\sqrt{r}\times \sqrt{r}$. Denote the first RO output measurements during enrolment as a vector random variable ${\tilde{X}}^{r}$ with a joint probability density function $f_{{\tilde{X}}^{r}}$. This model allows symbols of ${\tilde{X}}^{r}$ to have correlations. Furthermore, consider that the noise sequence ${\tilde{E}}^{r}$ is additive, and its random symbols have zero mean. Next, denote the second RO output measurement during reconstruction as ${\tilde{Y}}_{j}={\tilde{X}}_{j}+{\tilde{E}}_{j}$ for all j∈[1:r]. We next describe the new transform coding method that consists of the following steps:

1. Applying a 2D $\sqrt{r}\times \sqrt{r}$ orthogonal transformation to decorrelate *r* RO output measurements;
2. Modeling additive noise components and noiseless transform coefficients;
3. Equalizing the histograms by converting all the noiseless transform coefficients into realizations of the same random variable with the same mean and variance values, which allows one to reduce the hardware complexity by using the same scalar quantizer;
4. Quantization of each transform coefficient with scalar and uniform quantizers so that we have an almost uniformly distributed and independent and identically distributed binary sequence $X^{n}$ (or its noisy version $Y^{n}=X^{n}+E^{n}$) via Gray labeling, followed by bit concatenation. Note that we impose the QoSec constraint on the probability distribution fitted to the equalized transform coefficients in the last step.

### 4.1. Step 1: Applying the Orthogonal Transforms

The transformation decorrelates RO measurements ${\tilde{X}}^{r}$ to obtain transform coefficients ${\tilde{T}}^{r}$, which are then quantized by using scalar quantizers separately such that the security loss is negligible. This result follows, since if the transform coefficients are uncorrelated and jointly Gaussian distributed, they are mutually independent; so, it is common, e.g., in the image processing and digital watermarking literature, to use transforms for this purpose [34]. It is observed that neighboring RO outputs in an array are highly correlated [11]. Thus, to measure the decorrelation performance of the applied transforms, we use the decorrelation efficiency that is determined by the autocovariance matrices before and after transformation [35]. The Karhunen–Loève transform (KLT) achieves the maximum decorrelation efficiency for a large set of probability distributions. However, the computational complexity is high for computing the KLT. Low-complexity 2D $\sqrt{r}\;\times \;\sqrt{r}$ transforms that achieve high decorrelation efficiency were proposed in [12]. The proposed set of transforms included the DWHT. In [23] [Section 4.1], the extensions of the DWHT were obtained via an exhaustive search over all 4×4 orthogonal matrices with elements {−1,1}; then, larger matrices were constructed by applying Kronecker products of the 4×4 orthogonal matrices with other matrices *A* that preserved the orthogonality. For instance, if a matrix *A* with elements {−1,1} is orthogonal, i.e., we have $AA^{T}=I$, then the following matrices are also orthogonal [23]:

(11) $$\begin{array}{cc} \; & \left[\begin{array}{cc} A & A\\ A & \;-\;A \end{array}\right],\left[\begin{array}{cc} A & A\\ \;-\;A & A \end{array}\right],\left[\begin{array}{cc} A & \;-\;A\\ A & A \end{array}\right],\left[\begin{array}{cc} \;-\;A & A\\ A & A \end{array}\right],\\ {}[ & \begin{array}{cc} \;-\;A & \;-\;A\\ \;-\;A & A \end{array}],\left[\begin{array}{cc} \;-\;A & \;-\;A\\ A & \;-\;A \end{array}\right],\left[\begin{array}{cc} \;-\;A & A\\ \;-\;A & \;-\;A \end{array}\right],\left[\begin{array}{cc} A & \;-\;A\\ \;-\;A & \;-\;A \end{array}\right]. \end{array}$$

Thus, via exhaustive search, one can obtain 12288 orthogonal 2D transforms with size 16×16. These transforms do not require multiplication, and the decorrelation performance loss as compared to the DCT is negligible [12,23]. We define the transform, whose maximum error probability for the RO output dataset [36] over all transform coefficients is the smallest value among all obtained transforms, as the selected transform (ST). In Section 6, we thus apply the ST for RO PUF security and reliability analysis.

### 4.2. Step 2: Modeling Noise Components and Transform Coefficients

We applied one of the transforms in the set, obtained by computing the matrices in (11) for all 4×4 orthogonal matrices *A* with elements {−1,1}, to the RO measurements ${\tilde{X}}^{r}$ in the dataset [36] to obtain the transform coefficients ${\tilde{T}}^{r}$. In [23], it is shown that Gaussian distributions are good fits for all transform coefficients, which does not take into account that RO output realizations are positive real numbers within a finite range. Truncated Gaussian distributions were thus fitted to the used transform coefficients ${\tilde{T}}_{j}$, i.e., only for j∈[2:r] because the first coefficient ${\tilde{T}}_{1}$ is equal to a fraction of the average oscillation frequency over all ROs that can be estimated reliably by using the other transform coefficients [9]. We applied maximum-likelihood estimation methods to obtain unbiased variance and mean values for the fitted distributions. Furthermore, the finite range for each coefficient was fixed by using the transform coefficients that were obtained from the RO dataset in [36]. Note that the same transform was applied both to ${\tilde{X}}^{r}$ and ${\tilde{Y}}^{r}$, such that the transform coefficients computed from ${\tilde{Y}}^{r}$ were noisy transform coefficients denoted as $\left({\tilde{T}}_{j}+{\tilde{N}}_{j}\right)$ for all j∈[2:r], in which the noise components ${\tilde{N}}_{j}$ were zero-mean Gaussian distributed and mutually independent as well as independent of ${\tilde{T}}^{r}$.

### 4.3. Step 3: Equalizing Histograms

We applied a histogram equalization step, as proposed in [37], to convert each transform coefficient ${\tilde{T}}_{j}$ into a standard normal distribution, as one can model all transform coefficients as a Gaussian distribution with mean $\mu_{{\tilde{T}}_{j}}\neq 0$ and variance $\sigma_{{\tilde{T}}_{j}}^{2}\neq 1$ [37]. Consider that an original Gaussian distribution that is fitted to a transform coefficient has a mean of $\mu_{{\tilde{T}}_{j,\mathrm{orig}}}$ and a variance of $\sigma_{{\tilde{T}}_{j,\mathrm{orig}}}^{2}$, such that we can uniquely obtain the parameters of the corresponding truncated Gaussian distribution by bounding its range from both below and above [38]. We denote the mean and variance of the truncated Gaussian distribution as $\mu_{{\tilde{T}}_{j,\mathrm{trun}}}$ and $\sigma_{{\tilde{T}}_{j,\mathrm{trun}}}^{2}$, respectively. Therefore, to apply the histogram equalization step we subtracted the value $\mu_{{\tilde{T}}_{j,\mathrm{trun}}}$ and then divided the result by $\sigma_{{\tilde{T}}_{j,\mathrm{trun}}}$ for each realization ${\tilde{T}}_{j}={\tilde{t}}_{j}$. We denote the resulting equalized transform coefficient as ${\bar{\tilde{T}}}_{j}$ and the resulting additive zero-mean mutually-independent Gaussian noise component with variance $\sigma_{{\bar{\tilde{N}}}_{j}}^{2}$ as ${\bar{\tilde{N}}}_{j}$, respectively, for all j∈[2:r].

### 4.4. Step 4: Reliable Bit Extraction with QoSec Guarantees by Quantizing Noisy Coefficients

Consider that we extract $m_{j}\geq 0$ uniformly distributed and mutually independent bits from an equalized transform coefficient ${\bar{\tilde{T}}}_{j}$ for j∈[2:r], such that we can use the FCS with almost uniformly distributed and independent and identically distributed binary sequences $X^{n}$. For the *j*-th uniform scalar quantizer, we denote the quantization boundaries as $b_{j,0},b_{j,1},\ldots ,b_{j,2^{m_{j}}}$, where we have $b_{j,0}$ and $b_{j,2^{m_{j}}}$ as the lower and upper bounds on the range of the truncated Gaussian distributed ${\bar{\tilde{T}}}_{j}$, respectively. For all $k_{j}\in \left[1\;:\;\left(2^{m_{j}}\;-\;1\right)\right]$ and j∈[2:r], we assigned the quantiles of the *j*-th equalized and truncated Gaussian distribution to the quantization boundaries, i.e., we obtained

(12) $$\begin{array}{c} b_{j,k_{j}}\;=\;Q^{-1}\left(Q\left(b_{j,0}\right)\;\cdot \;\left(1\;-\;\frac{k_{j}}{2^{m_{j}}}\right)+Q\left(b_{j,2^{m_{j}}}\right)\;\cdot \;\frac{k_{j}}{2^{m_{j}}}\right). \end{array}$$

Given a realization ${\bar{\tilde{t}}}_{j}$ or its noisy version $\left({\bar{\tilde{t}}}_{j}\;+\;{\bar{\tilde{n}}}_{j}\right)$, the quantizer in (12) outputs $k_{j}$ if $b_{j,(k_{j}-1)}\;<\;{\bar{\tilde{t}}}_{j}\;\leq \;b_{j,k_{j}}$. Furthermore, since each additive noise component ${\bar{\tilde{N}}}_{j}$ has zero mean, we appled Gray labeling to map each $k_{j}$ to a bit sequence of size $m_{j}$ for all j∈[2:r]. This follows since Gray labeling results in only one bit flip if a noisy transform coefficient is quantized into a neighboring quantization interval.

### 4.5. Step 5: Bit Sequence Concatenation

Finally, we concatenated the bit sequences extracted from all the used transform coefficients to obtain a bit sequence that referred to $X^{n}$ if the first RO measurements ${\tilde{X}}^{r}$ were given as input and to $Y^{n}$ if the second RO measurements ${\tilde{Y}}^{n}={\tilde{X}}^{n}+{\tilde{E}}^{n}$ were given as input, respectively. Thus, we obtained a sequence $x^{n}$ via the concatenation of the bit sequences extracted from (r−1) equalized transform coefficients; so, we have $n\;=\;\sum_{j=2}^{r}m_{j}$.

## 5. Analysis for QoSec Guarantees

Consider that we observe a transform coefficient realization at a quantization boundary, i.e., ${\bar{\tilde{t}}}_{j}=b_{j,k_{j}}$ for some $k_{j}\in \left[1:\left(2^{m_{j}}\;-\;1\right)\right]$ and j∈[2:r]. For this realization, the error probability with 1-bit quantization is 0.5. Thus, the reliable reconstruction of the corresponding bit sequence is not possible; see [39,40] for similar discussions with different design metrics and without QoSec guarantees. Therefore, to provide reliability guarantees to each RO PUF output, one should eliminate unreliable realizations before quantization, i.e., the transform coefficient realizations that are spatially close to the quantization boundaries. We thus propose to eliminate the realizations that are in the range

(13) $$\begin{array}{c} {\bar{\tilde{t}}}_{j}\in \left(\left(b_{j,k_{j}}\;-\;\delta /2\right),\;\left(b_{j,k_{j}}\;+\;\delta /2\right)\right] \end{array}$$

for all $k_{j}\in \left[1:\left(2^{m_{j}}\;-\;1\right)\right]$ and j∈[2:r] and for some fixed δ≥0; so, the parameter δ is a *QoSec parameter* for all PUF outputs used for SK agreement with the FCS. We denote the ratio of the eliminated realizations vs. all the realizations for all j∈[2:r] as

(14) $$\begin{array}{c} \gamma_{j}\left(\delta \right)=\frac{\sum_{k_{j}=1}^{\left(2^{m_{j}}\;-\;1\right)}\left(Q\left(b_{j,k_{j}}\;-\;\frac{\delta }{2}\right)\;-\;Q\left(b_{j,k_{j}}\;+\;\frac{\delta }{2}\right)\right)}{Q\left(b_{j,0}\right)-Q\left(b_{j,2^{m_{j}}}\right)}. \end{array}$$

When δ is fixed, the percentage $\beta_{j}$ of realizations ${\bar{\tilde{t}}}_{j}$ that can be used for the SK agreement is defined as the *secure manufacturing yield* and is calculated as

(15) $$\begin{array}{c} \beta_{j}\left(\delta \right)\;=\;100\;\cdot \;\left(1\;-\;\gamma_{j}\left(\delta \right)\right) \end{array}$$

for all j∈[2:r], which decreases for increasing δ. The worst case error probability then decreases from 0.5 to $Q\left(\delta /2\sigma_{{\bar{\tilde{N}}}_{j}}\right)$ for 1-bit quantization; so, δ represents a worst case reliability guarantee.

We next illustrate that the error probabilities for different bits extracted from the same coefficient are dependent, i.e., the channel $P_{Y|X}^{n}$ has memory. This result proves that the FCS, which requires $P_{Y|X}^{n}$ to be memoryless, can be improved by taking the memory in the channel into account. Suppose that, e.g., m=2 bits are extracted from $\bar{\tilde{T}}$ by applying a binary-reflected Gray labeling, i.e., the quantization intervals are mapped to “00”, “01”, “11”, and “10” in the given order. We obtain

(16) $$\begin{array}{cc} \; & \mathrm{Pr}\left[\left\{1\mathrm{st}\;\mathrm{bit}\;\mathrm{is}\;\mathrm{in}\;\mathrm{error}\right\}|\bar{\tilde{t}}\;\right]\cdot \left(Q\left(b_{0}\right)-Q\left(b_{2^{m}}\right)\right)\cdot \left(1-\gamma \left(\delta \right)\right)\\ \; & \;\;\;\;\;\;\;=\left\{\begin{array}{cc} Q\left(\frac{b_{2}-\bar{\tilde{t}}}{\sigma_{\bar{\tilde{N}}}}\right) & \mathrm{if}\;\;\bar{\tilde{t}}\in \left[b_{0},\;\left(b_{2}-\frac{\delta }{2}\right)\right]\\ Q\left(\frac{\bar{\tilde{t}}-b_{2}}{\sigma_{\bar{\tilde{N}}}}\right) & \mathrm{if}\;\;\bar{\tilde{t}}\in \left(\left(b_{2}+\frac{\delta }{2}\right),\;b_{4}\right] \end{array}\right. \end{array}$$

and

(17) $$\begin{array}{ccc} \; & \mathrm{Pr}\left[\left\{2\mathrm{nd}\;\mathrm{bit}\;\mathrm{is}\;\mathrm{in}\;\mathrm{error}\right\}|\bar{\tilde{t}}\;\right]\cdot \left(Q\left(b_{0}\right)-Q\left(b_{2^{m}}\right)\right)\cdot \left(1-\gamma \left(\delta \right)\right) & \\ \; & \;\;\;\;=\left\{\begin{array}{cc} Q\left(\frac{b_{1}-\bar{\tilde{t}}}{\sigma_{\bar{\tilde{N}}}}\right)-Q\left(\frac{b_{3}-\bar{\tilde{t}}}{\sigma_{\bar{\tilde{N}}}}\right) & \mathrm{if}\;\bar{\tilde{t}}\in \left[b_{0},\;\left(b_{1}-\frac{\delta }{2}\right)\right]\\ Q\left(\frac{\bar{\tilde{t}}-b_{1}}{\sigma_{\bar{\tilde{N}}}}\right)+Q\left(\frac{b_{3}-\bar{\tilde{t}}}{\sigma_{\bar{\tilde{N}}}}\right) & \mathrm{if}\;\bar{\tilde{t}}\in \left(\left(b_{1}+\frac{\delta }{2}\right),\;\left(b_{3}-\frac{\delta }{2}\right)\right]\\ Q\left(\frac{b_{1}-\bar{\tilde{t}}}{\sigma_{\bar{\tilde{N}}}}\right)-Q\left(\frac{b_{3}-\bar{\tilde{t}}}{\sigma_{\bar{\tilde{N}}}}\right) & \mathrm{if}\;\bar{\tilde{t}}\in \left(\left(b_{3}+\frac{\delta }{2}\right),\;b_{4}\right]. \end{array}\right. \end{array}$$

Applying the law of total probability and Bayes’ theorem to $\mathrm{Pr}[\left\{1\mathrm{st}\;\mathrm{bit}\;\mathrm{is}\;\mathrm{in}\;\mathrm{error}\right\}|\bar{\tilde{t}}]$ (or $\mathrm{Pr}[\left\{2\mathrm{nd}\;\mathrm{bit}\;\mathrm{is}\;\mathrm{in}\;\mathrm{error}\right\}|\bar{\tilde{t}}]\;$), we obtain the formula for the probability of the first (or second) bit being erroneous conditioned on the event that the equalized transform coefficient $\bar{\tilde{t}}$ falls into the corresponding quantization interval. Since closed form expressions do not seem to exist for these probabilities, we computed them numerically for various parameters and observe that the multiplication of these two marginal probabilities was generally not equal to the corresponding joint probability. Therefore, it is a numerically computed proof on which errors in the first and second bits conditioned on a quantization interval, which determines the mapped bit sequence, are dependent, i.e., the channel $P_{Y|X}$ is not memoryless; so, it is not optimal to use the FCS. We remark that a memoryless channel model can still be used as a pessimistic reference model for which correlations cannot be taken advantage of, which follows since the FCS treats secrecy and reliability separately.

Now, we define an alternative reliability metric $P_{c}$, called *correctness probability*, as the probability that all extracted bits are correct, as proposed in [41]. This metric is a conservative metric, and we use it below in combination with the FCS. For an equalized transform coefficient $\bar{\tilde{T}}$ with QoSec parameter δ, we have the correctness probability

(18) $$\begin{array}{cc} \; & P_{c}\left(\delta \right)\cdot \left(Q\left(b_{0}\right)-Q\left(b_{2^{m}}\right)\right)\cdot \left(1-\gamma \left(\delta \right)\right)\\ \; & \;\;\;\;=\int_{b_{0}}^{\left(b_{1}-\delta /2\right)}\left[\;Q\left(\frac{b_{0}\;-\;\bar{\tilde{t}}}{\sigma_{\bar{\tilde{N}}}}\right)\;-\;Q\left(\frac{b_{1}\;-\;\bar{\tilde{t}}}{\sigma_{\bar{\tilde{N}}}}\right)\;\right]f_{\bar{T}}\left(\bar{\tilde{t}}\right)d\bar{\tilde{t}}\\ \; & \;\;\;\;\;+\sum_{k=1}^{\left(2^{m}-2\right)}\int_{\left(b_{k}+\delta /2\right)}^{\left(b_{\left(k+1\right)}-\delta /2\right)}\left[\;Q\left(\frac{b_{k}\;-\;\bar{\tilde{t}}}{\sigma_{\bar{\tilde{N}}}}\right)\;-\;Q\left(\frac{b_{\left(k+1\right)}\;-\;\bar{\tilde{t}}}{\sigma_{\bar{\tilde{N}}}}\right)\;\right]f_{\bar{T}}\left(\bar{\tilde{t}}\right)d\bar{\tilde{t}}\\ \; & \;\;\;\;\;+\int_{\left(b_{\left(2^{m}-1\right)}+\delta /2\right)}^{b_{2^{m}}}\;\left[Q\left(\frac{b_{\left(2^{m}-1\right)}\;-\;\bar{\tilde{t}}}{\sigma_{\bar{\tilde{N}}}}\right)\;-\;Q\left(\frac{b_{2^{m}}\;-\;\bar{\tilde{t}}}{\sigma_{\bar{\tilde{N}}}}\right)\;\right]f_{\bar{T}}\left(\bar{\tilde{t}}\right)d\bar{\tilde{t}}. \end{array}$$

where $\bar{T}$ is a random variable distributed according to a standard Gaussian distribution with the probability density function $f_{\bar{T}}$.

## 6. QoSec Guarantee Effects on RO PUFs

**Figure 3:**
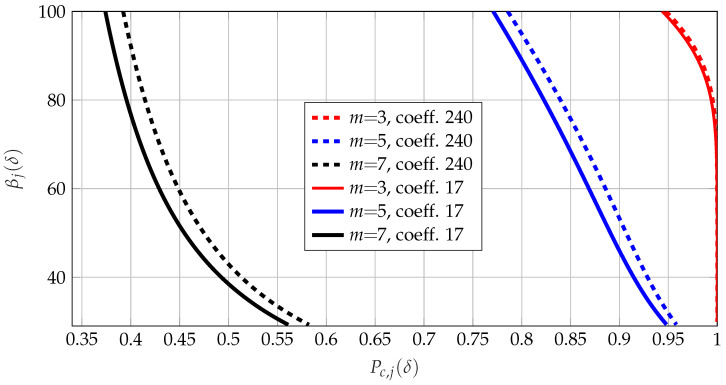
Correctness probability $P_{c,j}\left(\delta \right)$ vs. secure manufacturing yield $\beta_{j}\left(\delta \right)$ with the ST applied to 16×16 RO arrays from the dataset in [36]. We achieve $\left(\beta_{j}=100,\delta =0\right)$, and $\beta_{j}$ decreases with increasing δ. In row ⌈j/16⌉ and column (jmod16), we have the *j*-th transform coefficient.

We used the public RO output dataset [36], consisting of 100 noisy measurements of 32×16 RO output arrays obtained from 193 different devices, but we considered only the upper part of the array, such that we have $\sqrt{r}=16$ to apply the transform coding steps described in Section 4. In Step 1, we applied the ST to the 16×16 RO array. Applying Steps 2–4, we computed the secure manufacturing yield $\beta_{j}\left(\delta \right)$ by using (15) and $P_{c,j}\left(\delta \right)$ from (18), respectively. We plot in Figure 3 the effects of δ on tuples $\left(P_{c,j},\;\beta_{j}\right)$ for two randomly-chosen transform coefficients that were uniformly quantized by using three different bit sequence lengths, i.e., $m_{j}\;=\;3,5,7$.

When δ increased, the percentage of realizations that could be used decreased, whereas the correctness probability increased as depicted in Figure 3. We define the minimum quantization interval length as

(19) $$\begin{array}{c} ∆b_{j}=\min_{k_{j}\in \left[0:2^{m_{j}}-1\right]}\left(b_{j,(k_{j}+1)}\;-\;b_{j,k_{j}}\right) \end{array}$$

for all j∈[2:r]. The allowed range of values for δ was chosen to be $0\;\leq \;\delta \;\leq \;∆b_{j}$ for each coefficient, since at its maximum value, at least half of the realizations were removed, and further removal might not be practical. We observe from Figure 3 that for most transform coefficients the decreasing pattern of $\beta_{j}$ with respect to $P_{c,j}$ for increasing δ was different for small, medium, and large numbers $m_{j}$ of extracted bits. Thus, it seems difficult to obtain a general algorithm that provides optimal operation points in terms secrecy, reliability, QoSec, code rate, etc. Therefore, we next extended the thresholding approaches proposed in [12,41] that impose thresholds only on $P_{c,j}$.

### 6.1. Proposed Joint Thresholding Approach for QoSec Guarantees

First, we assumed that a lower bound $\bar{\delta }\geq 0$ was imposed on the QoSec parameter δ, which could be imposed, for instance, due to the data privacy regulations. Next, we supposed that a linear ECC C could correct all error patterns in up to $\bar{r}\geq 1$ transform coefficients; thus, a lower bound ${\bar{P}}_{c}\left(\delta \right)$ on each correctness probability $P_{c,j}\left(\delta_{j}\right)$ for j∈[2:r] was determined by the block error probability $P_{e}$ such that

(20) ∑j¯=(r¯+1)(r−1)(r−1)j¯·(1−P¯c(δ))j¯·P¯c(δ)r−1−j¯≤Pe,

where ${\bar{P}}_{c}\left(\delta \right)$ is the minimum probability that satisfies the inequality. Furthermore, we assume that a chip manufacturer determines a lower bound $\bar{\beta }$ on each $\beta_{j}$ as a practical manufacturing constraint. The lower bound $\bar{\beta }$ corresponds to an upper bound ${\bar{\bar{\delta }}}_{j}$ on δ for all j∈[2:r], which follows from (14) and (15). Then, for the *j*-th transform coefficient, the maximum number of bits that satisfies both thresholds simultaneously is assigned to $m_{j}$, and we choose the value $\delta_{j}={\bar{\bar{\delta }}}_{j}$ that corresponds to an operation point $\left(P_{c,j}\left(\delta_{j}\right),\beta_{j}\left(\delta_{j}\right)\right)$. We can then guarantee a QoSec parameter of δ that is chosen as the minimum $\delta_{j}$ over all transform coefficients, which provides a guarantee for the worst case security and reliability of all sequences extracted from all PUFs; see also Remark 1.

**Remark** **1.** *Choosing $\delta =\min_{j\in [2:r]}\delta_{j}$ does not necessarily provide the same security guarantee for all bit sequences extracted from all PUFs because the noise variances for each transform coefficient can be different. However, we observe that the noise variances for all transform coefficients were similar for the transform coding method applied to the considered ROs; so, the security guarantee follows for this setup.*

To apply the proposed approach, we chose the number of bits $m_{j}\geq 0$ extracted from each transform coefficient j∈[2:r], such that all corresponding operation points $\left(P_{c,j}\left(\delta_{j}\right),\beta_{j}\left(\delta_{j}\right)\right)$ simultaneously satisfied

1. $\delta \geq \bar{\delta }$;
2. $P_{c,j}\left(\delta_{j}\right)\geq {\bar{P}}_{c}\left(\delta \right)$; and
3. $\beta_{j}\left(\delta_{j}\right)\geq \bar{\beta }$.

Recall that the calculation of the lower bound ${\bar{P}}_{c}\left(\delta \right)$ that should satisfy (20) assumes that the number $\bar{r}$ of transform coefficients that a given ECC C should correct is known, which is not the case in practice. To determine the parameters of an ECC C that can correct $\bar{r}$ transform coefficients, we first calculate the blocklength of the ECC, i.e., the total number of extracted bits after bit concatenation, which is $n=\sum_{j=2}^{r}m_{j}$. Moreover, we sort the numbers $m_{j}$ of bits extracted from all transform coefficients in a descending order, i.e., $m_{j}^{\prime }\geq m_{j+1}^{\prime }$ for all j∈[2:r]; so, the ECC C must correct all bit error patterns with up to $e\left(\bar{r}\right)=\sum_{j=2}^{\left(\bar{r}+1\right)}m_{j}^{\prime }$ errors. Using a block code with minimum distance $d_{min}\geq 2e\left(\bar{r}\right)+1$, this constraint can be satisfied. Thus, our joint thresholding approach provides a practical method to design RO PUFs with QoSec guarantees.

### 6.2. Effects of QoSec Guarantees on ECC Design

We next illustrate the effects of providing QoSec guarantees. First, we impose the condition $m_{j}=1$ for all j∈[2:n] to simplify the analysis, which also has the side benefit that the error components are then not correlated, as discussed in Section 5. Thus, we have n=255 and $e\left(\bar{r}\right)=\bar{r}$ such that a block code with $d_{min}=\left(2\bar{r}+1\right)$ can be used. We impose a practical block error probability constraint such that $P_{e}\leq 10^{-9}$, as in [12,42]. We then consider a set of lower bounds

(21) $$\begin{array}{c} {\bar{\delta }}_{j}=\left\{0,\;0.01,\;0.03,\;0.05\right\}\times ∆b_{j}, \end{array}$$

where $∆b_{j}$ is as defined in (19) above, on the QoSec parameter δ that represents different levels of security, which can be considered to be imposed by a legal entity to ensure IoT device security. Furthermore, we assume that the manufacturer cannot afford to have a lower manufacturing yield than the secure manufacturing yield determined by the legally imposed lower bound on the QoSec parameter, i.e., $\delta_{j}=\delta =\bar{\delta }$ for all j∈[2:r]. Then, we obtain the secure manufacturing yields of

(22) $$\begin{array}{c} \beta_{j}\left(\delta \right)=\left\{100,\;97.951,\;93.850,\;89.784\right\}, \end{array}$$

for all j∈[2:r] and for $\delta ={\bar{\delta }}_{j}$ values listed in (21), respectively. Moreover, we have the average correctness probabilities of {0.990965,0.997468,0.999935,0.999999} averaged over all transform coefficients for ${\bar{\delta }}_{j}$ values given in (21). We approximate the corresponding measurement channels as BSCs with crossover probabilities

(23) $$\begin{array}{cc} \; & p=\{9.035\times 10^{-3},\;2.532\times 10^{-3},\;6.541\times 10^{-5},\;6.918\times 10^{-7},\} \end{array}$$

which are obtained by subtracting the average correctness probabilities from 1. Thus, by applying Theorem 2, while ignoring the O(1/n) term in (7), we obtain the finite-length results that show that code dimensions

(24) $$\begin{array}{c} \left\lfloor nR_{s}\right\rfloor =\left\{208,\;203,\;186,\;180\right\} \end{array}$$

can be achieved by the crossover probabilities in (23), respectively. We also combine the corresponding results of (21)–(24) in Table 1, given on the next page, for convenience. Note that if we consider that the SK *S* that is bound to the PUF outputs in Figure 2 is used for symmetric cryptography, e.g., for advanced encryption standard (AES) with 128 bits of SK, this corresponds to a code dimension of 128 bits. The code dimensions given in (24) that can be achieved for n=255 and $P_{e}=10^{-9}$ suffice to use the SK *S* with AES-128. Thus, the results in (22) and (24) illustrate that providing QoSec guarantees to PUFs does not cause a significant performance degradation.

## 7. Conclusions

In this work, we have developed realistic models for transformed RO outputs by fitting truncated distributions to them. After illustrating that reliability and security cannot be guaranteed for each PUF device by using the state-of-the-art methods, we proposed a new transform coding method that takes a QoSec parameter into consideration to extract SKs from PUFs. Our joint thresholding approach provides QoSec guarantees for target SK sizes and block error probabilities. Using finite-length bounds on the region of all (secret-key, privacy-leakage) rate pairs that are achievable by using the FCS, we showed that QoSec guarantees can be given for each extracted bit of all PUF devices without a significant performance degradation. In future, we plan to analyze the effects of random and systematic variations in digital circuit outputs separately, rather than modeling the total effect as an additive noise component, to provide a more accurate PUF model.

## Figures and Tables

**Table 1:** Effects of providing QoSec guarantees.

${\bar{\delta }}_{j}/∆b_{j}$	0	0.01	0.03	0.05
$\beta_{j}\left(\delta \right)$	100	97.951	93.850	89.784
p	$9.035\times 10^{-3}$	$2.532\times 10^{-3}$	$6.541\times 10^{-5}$	$6.918\times 10^{-7}$
$\left\lfloor nR_{s}\right\rfloor$	208	203	186	180

## Data Availability

Not applicable.

## References

[1] Gassend B. (2003). Physical Random Functions. Master’s Thesis.

[2] Pappu R. (2001). Physical One-way Functions. Ph.D. Thesis.

[3] Devadas S., Gassend B., Clarke D., Van Dijk M. (2007). Controlling Access to Device-Specific Information. US Patent.

[4] Günlü O. (2018). Key Agreement with Physical Unclonable Functions and Biometric Identifiers. Ph.D. Thesis.

[5] Kusters L., Günlü O., Willems F.M. Zero Secrecy Leakage for Multiple Enrollments of Physical Unclonable Functions. Proceedings of the 2018 Symposium on Information Theory and Signal Processing in the Benelux.

[6] Guajardo J., Kumar S.S., Schrijen G.J., Tuyls P. (2007). FPGA Intrinsic PUFs and Their Use for IP Protection.

[7] Suh G.E., Devadas S. Physical Unclonable Functions for Device Authentication and Secret Key Generation. Proceedings of the ACM Design Automation Conf..

[8] Bloch M., Günlü O., Yener A., Oggier F., Poor H.V., Sankar L., Schaefer R.F. (2021). An Overview of Information-Theoretic Security and Privacy: Metrics, Limits and Applications. IEEE J. Sel. Areas Inf. Theory.

[9] Günlü O. (2013). Design and Analysis of Discrete Cosine Transform Based Ring Oscillator Physical Unclonable Functions. Master’s Thesis.

[10] Böhm C., Hofer M. (2012). Physical Unclonable Functions in Theory and Practice.

[11] Merli D., Stumpf F., Eckert C. Improving the quality of ring oscillator PUFs on FPGAs. Proceedings of the ACM Workshop Embedded Sys. Security.

[12] Günlü O., Kernetzky T., İşcan O., Sidorenko V., Kramer G., Schaefer R.F. (2018). Secure and Reliable Key Agreement with Physical Unclonable Functions. Entropy.

[13] Hospodar G., Maes R., Verbauwhede I. Machine learning attacks on 65nm Arbiter PUFs: Accurate modeling poses strict bounds on usability. Proceedings of the IEEE International Workshop on Information Forensics and Security.

[14] Dodis Y., Ostrovsky R., Reyzin L., Smith A. (2008). Fuzzy extractors: How to generate strong keys from biometrics and other noisy data. SIAM J. Comput..

[15] Juels A., Wattenberg M. A fuzzy commitment scheme. Proceedings of the ACM Conference on Computer and Communication Security.

[16] Günlü O., Trifonov P., Kim M., Schaefer R.F., Sidorenko V. Randomized Nested Polar Subcode Constructions for Privacy, Secrecy, and Storage. Proceedings of the International Symposium on Information Theory and Its Applications.

[17] Günlü O., İşcan O., Sidorenko V., Kramer G. (2019). Code Constructions for Physical Unclonable Functions and Biometric Secrecy Systems. IEEE Trans. Inf. Forensics Secur..

[18] Ignatenko T., Willems F.M.J. (2009). Biometric systems: Privacy and secrecy aspects. IEEE Trans. Inf. Forensics Secur..

[19] Maurer U.M. (1993). Secret Key Agreement by Public Discussion from Common Information. IEEE Trans. Inf. Theory.

[20] Ignatenko T., Willems F.M.J. (2010). Information Leakage in Fuzzy Commitment Schemes. IEEE Trans. Inf. Forensics Secur..

[21] Ahlswede R., Csiszár I. (1993). Common Randomness in Information Theory and Cryptography - Part I: Secret Sharing. IEEE Trans. Inf. Theory.

[22] Günlü O., İşcan O. DCT Based Ring Oscillator Physical Unclonable Functions. Proceedings of the IEEE International Conference on Acoustics, Speech and Signal Processing, ICASSP 2014.

[23] Günlü O., Schaefer R.F. Low-complexity and Reliable Transforms for Physical Unclonable Functions. Proceedings of the IEEE International Conference on Acoustics, Speech and Signal Processing, ICASSP 2020.

[24] Wayman J., Jain A., Maltoni D., (Eds) D.M. (2005). Biometric Systems: Technology, Design and Performance Evaluation.

[25] Campisi P. (2013). Security and Privacy in Biometrics.

[26] de Groot J., Škoric B., Vreede N.D., Linnartz J.P. (2012). Information leakage of continuous-source zero secrecy leakage helper data schemes. Citeseer Gen.

[27] Etemoglu A.O., Cuperman V. (2003). Structured vector quantization using linear transforms. IEEE Trans. Signal Process..

[28] Li N., Zhang Y., Kuo C.C.J. Explainable Machine Learning Based Transform Coding for High Efficiency Intra Prediction. Arxiv.org/abs/2012.11152.

[29] Günlü O., Schaefer R.F., Poor H.V. Quality of Service Guarantees for Physical Unclonable Functions. Proceedings of the IEEE Int. Workshop Inf. Forensics Security.

[30] Mandal M.K., Sarkar B.C. (2010). Ring oscillators: Characteristics and Applications. Indian J. Pure Appl. Phys..

[31] Yin C.E., Qu G. Improving PUF security with regression-based distiller. Proceedings of the Automation Conference 2013, DAC ’13.

[32] Günlü O., Schaefer R.F., Kramer G. Private Authentication with Physical Identifiers Through Broadcast Channel Measurements. Proceedings of the IEEE Information Theory Workshop will be held in Visby.

[33] Polyanskiy Y., Poor H.V., Verdú S. (2010). Channel Coding Rate in the Finite Blocklength Regime. IEEE Trans. Inf. Theory.

[34] Wang R. (2012). Introduction to Orthogonal Transforms: With Applications in Data Processing and Analysis.

[35] Ohm J.R. (2015). Multimedia Signal Coding and Transmission.

[36] Maiti A., Casarona J., McHale L., Schaumont P. A Large Scale Characterization of RO-PUF. Proceedings of the IEEE International Symposium on Hardware-Oriented Security and Trust (HOST).

[37] Günlü O., İşcan O., Sidorenko V., Kramer G. Reliable Secret-key Binding for Physical Unclonable Functions with Transform Coding. Proceedings of the 2016 IEEE Global Conference on Signal and Information Processing.

[38] Johnson N.L., Kotz S., Balakrishnan N. (1994). Continuous Univariate Distributions.

[39] Voloshynovskiy S., Koval O., Holotyak T., Beekhof F. Privacy enhancement of common randomness based authentication: Key rate maximized case. Proceedings of the IEEE International Workshop on Information Forensics and Security, WIFS 2009.

[40] Voloshynovskiy S., Koval O., Holotyak T., Beekhof F., Farhadzadeh F. Privacy amplification of content identification systems based on fingerprint bit reliability. Proceedings of the IEEE International Workshop on Information Forensics and Security.

[41] Günlü O., Belkacem A., Geiger B.C. Secret-key Binding to Physical Identifiers with Reliability Guarantees. Proceedings of the IEEE International Conference on Communications.

[42] Maes R., Herrewege A.V., Verbauwhede I. (2012). PUFKY: A fully functional PUF-based cryptographic key generator. Cryptographic Hardware Embedded Sys..

